# Novel MAXPOWER biological antibacterial liquid for eradicating oral *Helicobacter pylori*

**DOI:** 10.1186/s12879-024-09424-8

**Published:** 2024-05-29

**Authors:** Yongkang Lai, Xiaoyang Dong, Yingxiao Song, Jiulong Zhao, Yiqi Du, Zhaoshen Li

**Affiliations:** 1 Department of Gastroenterology, Changhai Hospital, Naval Medical University, 168 Changhai Road, Yangp u District, Shanghai, 200433 China; 2 Department of Gastroenterology, Ganzhou People’s Hospital Affiliated to Nanchang University, Ganzhou, 341000 China; 3 National Clinical Research Center for Digestive Diseases, Changhai Hospital, Naval Medical University, Shanghai, China; 4 National key laboratory of Immunity and inflammation, Naval Medical University, Shanghai, China; 5 Changhai Clinical Research Unit, Changhai hospital, Naval Medical University, Shanghai, China

**Keywords:** Oral *Helicobacter pylori*, Antibiotic-free, MAXPOWER Biological Antibacterial Liquid, Randomized controlled trials, Antibacterial

## Abstract

**Background:**

Eradication of oral *Helicobacter pylori* (*H. pylori*) not only reduces the infection rate from the transmission route but also improves the success rate of intragastric eradication. MAXPOWER Biological Bacteriostatic Liquid, developed in our previous work, is a composite biological preparation with strong antibacterial ability and unique antibacterial mechanism. The present study evaluated the efficacy of the MAXPOWER biocontrol solution on *H. pylori* and its success rate in eradicating oral *H. pylori* in clinical patients.

**Methods:**

Live-dead cell staining and hemolysis test were used to evaluate the cellular safety of MAXPOWER biocontrol solution; plate spreading, live-dead bacterial staining, and scanning electron microscopy methods were used to evaluate its antimicrobial effect against *H. pylori*. Transcriptomics was used to analyze the changes in *H. pylori* genes before and after treatment. After seven days of gavage treatment, H&E staining and mice feces were collected for 16SrDNA sequencing to evaluate the animals’ safety. Oral *H. pylori-*positive patients were randomized to be given a placebo and MAXPOWER Bio-Bacteriostatic Liquid gargle for seven days to evaluate the effect on oral *H. pylori* eradication.

**Results:**

In vitro tests demonstrated that this product has excellent biocompatibility and hemocompatibility and can effectively eradicate oral *H. pylori. In vivo* tests further showed that it has good biosafety and virtually no adverse effect on intestinal microflora. Transcriptomics analysis revealed that it kills *H. pylori* cells mainly by disrupting their cell membranes and metabolism. Additionally, the results of randomized controlled trials on humans disclosed that the oral *H. pylori* eradication rates achieved by MAXPOWER Biological Antibacterial Liquid were 71.4% and 78.9% according to the intention-to-treat and the per-protocol analysis, respectively.

**Conclusion:**

MAXPOWER Biological Antibacterial Liquid is both safe and efficacious in the eradication of oral *H. pylori*.

**Trial registration:**

This study was retrospectively registered in the ClinicalTrials.gov Trial Registry on 21/09/2023 (NCT06045832).

**Supplementary Information:**

The online version contains supplementary material available at 10.1186/s12879-024-09424-8.

## Background

*Helicobacter pylori (H. pylori)* is a gram-negative bacterium that has infected nearly half the global population and is closely associated with various gastrointestinal (GI) and extra-GI diseases, such as gastric cancer, peptic ulcers, chronic gastritis, and iron-deficiency anemia [1, 2]. Early in the 1990s, Correa proposed for the first time that *H. pylori* infection was the initiating factor in the evolution of intestinal gastric cancer [3]. Not long after, the International Agency for Research on Cancer (IARC), a subsidiary of the World Health Organization (WHO), classified *H. pylori* as a Class I carcinogen [4]. Subsequent extensive clinical and basic research on Helicobacter pylori further confirmed its close association with gastric cancer. In 2022, chronic Helicobacter pylori infection was officially defined as a definite carcinogen [5, 6]. Thus, the eradication of *H. pylori* is of great clinical significance in achieving the goal of reducing or eliminating deaths associated with gastric cancer [7].

The current treatment regimen for *H. pylori* is a quadruple 10–14-d regimen containing two different antibiotics [1]. Although nearly 40 years of clinical experience and professional consensus have resulted in revised and updated *H. pylori* treatment recommendations and protocols, the overall eradication rate of *H. pylori* infection remains poor, and global *H. pylori* infection rates remain high [8]. In addition to the failure of *H. pylori* eradication due to antibiotic resistance, reinfection and recurrence are also problematic. A meta-analysis of 17 studies revealed that the annual *H. pylori* recurrence rate may be as high as 13% in developing countries [9]. Poor hygiene conditions that promote *H. pylori* transmission in the population via oral-oral or fecal-oral routes are an independent risk factor responsible for the recurrence of this infection [10]. Previous studies have isolated and cultured *H. pylori* from the vomit and saliva of infected individuals [11, 12]. Flores-Treviño et al. and Kadota et al. detected *H. pylori* nucleic acids in dental plaque and tooth decay [13, 14]. These discoveries suggest that *H. pylori* may reside in the oral cavity, which could serve as a potential bacterial “reservoir” contributing to its spread and recurrence. Additionally, our recent epidemiological study disclosed that *H. pylori* infection tends to cluster within families where it is transmitted by the oral route [15]. Family members infected with *H. pylori* are potential sources of the pathogen and may diffuse it to their kin through their eating and other living habits [2]. Therefore, maintaining good oral hygiene may help in mitigating oral infection and transmission, lower the risk of recurrence, and improve the successful eradication of gastric *H. pylori*.

However, currently, applied eradication regimens have low apparent efficacy against oral *H. pylori*. Studies have shown that *H. pylori* can still be detected in the oral cavities of patients who have undergone successful gastric eradication [12, 16]. Furthermore, the current *H. pylori* eradication regimens involve long-term antibiotic administration that causes side effects severe enough to reduce patient compliance and adherence. Thus, there is an urgent need for a novel therapeutic modality that targets oral *H. pylori*, is antibiotic-free and nonirritating, and maintains oral probiotic homeostasis.

**Fig. 1:**
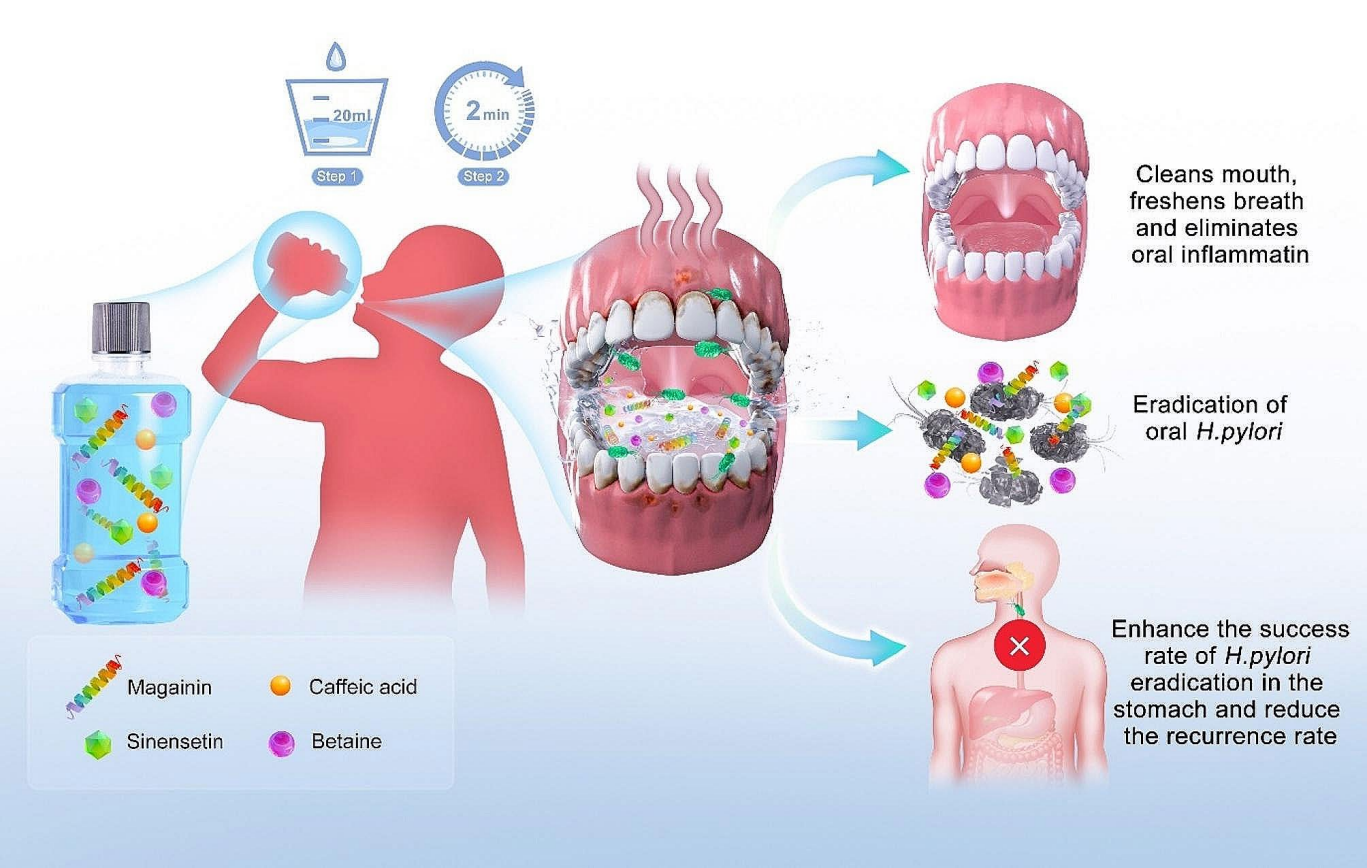
Schematic illustration of sterilized solution for the eradication of oral *Helicobacter pylori*

MAXPOWER Biological Antibacterial Liquid is a protein-active formulation developed in China using pure biotechnology. It adheres to the outer membrane of *H. pylori*, alters its permeability, and kills the cell. It is also differentially bacteriostatic. It targets and kills bacterial pathogens but has a much weaker inhibitory effect against probiotics. Thus, it is expected to stabilize the oral and gastric microecology while retaining its bactericidal efficacy. Moreover, MAXPOWER Biological Antibacterial Liquid can form nanoprotective biofilms at the interface between the surface of the outer membrane and the point at which the product makes contact with it. In this manner, MAXPOWER Biological Antibacterial Liquid temporarily inhibits microbial pathogens from colonizing it. Macromolecular antibacterial substances derived from pure biotechnology are unlikely to induce the pathogen resistance characteristic of antibiotic abuse, misuse, and overuse. Preliminary research revealed that MAXPOWER Biological Antibacterial Liquid has bactericidal efficacy comparable to that of antibiotics as well as good biological safety. Here, we constructed a MAXPOWER Biological Antibacterial Liquid-based mouthwash that targets oral *H. pylori* and evaluated the efficacy with which it eradicates this bacterial pathogen (Fig. 1).

## Methods

### Materials

Trimethoprim lactate (T9170), vancomycin (V8050), amphotericin B (A8251), and polymyxin B sulfate (P8350) were purchased from Sigma‒Aldrich Corp. (Shanghai, China). The Cell Counting Kit-8 (CK04), Urease Activity Assay Kit (No. BC4110), and Live/Dead Cell Staining Kit (No. 40747ES76) were purchased from Yeasen Biotechnology Co. Ltd. (Shanghai, China). The Urea Test Kit was purchased from Shandong Boan Biotech Co. Ltd. (Nanjing, China). The SYTO 9/PI Live/Dead Bacterial Double Stain Kit (No. MX4234-40T) was purchased from Shanghai Moukang Biotechnology Co. (Shanghai, China). The bicinchoninic acid (BCA) protein assay kit (No. 23,227) was purchased from Thermo Fisher Scientific Inc. (Waltham, MA, USA). The *H. pylori* strain was provided by the Department of Gastroenterology of the First Affiliated Hospital of Nanchang University (Jiangxi, China).

### Cell culture and *H. pylori* strains

Mouse fibroblasts (L929) were purchased from the Cell Bank of the Chinese Academy of Sciences (Beijing, China) and cultured in Dulbecco’s modified Eagle’s medium (DMEM)/F12 (Gibco, Grand Island, NY, USA) supplemented with 10% (v/v) fetal bovine serum (FBS) and 1% (w/v) penicillin/streptomycin in an incubator under a 5% CO_2_ atmosphere at 37 °C. The *H. pylori* strain CagA + ATCC43504 used in the present study was provided by Prof. Chuan Xie of the First Affiliated Hospital of Nanchang University. All *H. pylori* strains were stored at -80 °C. Before use, they were revived and cultured for two generations on blood agar plates containing 5% (v/v) sheep blood and 1% (w/v) mixed antibiotics under a 10% CO_2_ and 5% O_2_ atmosphere at 37 °C. The *H. pylori* liquid culture system comprised *Brucella* broth, 10% (v/v) FBS, and 0.5% (w/v) mixed antibiotics, and the cultures were incubated on microshakers.

### Mice

All animal experiments were conducted per the protocols approved by the Laboratory Animal Center of Changhai Hospital of Naval Medical University (Shanghai, China) (SYXK2020-0033) and are reported in accordance with ARRIVE guidelines. Kunming mice were purchased from the Shanghai Laboratory Animal Center (Shanghai, China) and maintained under specific pathogen-free (SPF) conditions at the Institute of Pancreatic Diseases of Shanghai Changhai Hospital (Shanghai, China). They were housed at ~ 55% relative humidity and 18–22 °C under a 12-h light/12-h dark cycle.

### In vitro cytocompatibility

In vitro cytocompatibility was assessed by Cell Counting Kit-8 (CCK-8) and live/dead cell staining assays. L929 cells were seeded in 96-well plates at a density of 10^4^/well. The cells were allowed to adhere overnight, and different concentrations of MAXPOWER biological antibacterial solution (original solution, 1:1, 1:2, 1:4, 1:8, 1:16) were added to the culture medium to explore the optimal concentration with cell safety. The cells were then incubated for 24 h, and each culture medium was replaced with 100 µL of 10% (w/v) CCK-8 dye solution per well. Incubation was then resumed for another 2 h. Cell viability was calculated by measuring the absorbance of each well at 450 nm (OD_450_) in a microreader (SpectraMax® i3; Molecular Devices LLC, San Jose, CA, USA). There were three replicate wells per group, and cell viability was calculated as shown in Eq. (1) below:

1 $$\begin{aligned}Cell\;viability \left(\%\right) & = (ODexperimental groups - ODblank groups)\\&\quad/(ODcontrol groups - ODblank groups) \times 100\%\end{aligned}$$

The live/dead cell staining assay was performed by coculturing the MAXPOWER Biological Antibacterial Liquid with the L929 cells and then replacing the medium with calcein AM and propidium iodide (PI) dyes at 37 °C for 30 min. The cells were then observed under an inverted-phase contrast microscope (DMIL LED, Leica Microsystems, Wetzlar, Germany).

### Hemolysis evaluation

Fresh blood was collected from the rats, centrifuged at 5,000 rpm for 3 min, and washed thrice with phosphate-buffered saline (PBS). Then, 2 mL of blood was diluted to 50 mL with saline, and 300 µL of the diluted blood was combined with 1.2 mL of saline solution containing various MAXPOWER Biological Antibacterial Liquid concentrations (original solution, 1:1, 1:2, 1:4, 1:8, 1:16). The negative and positive controls were prepared by mixing 300 µL erythrocytes with 1.2 mL saline and 1.2 mL deionized water, respectively. The mixtures were incubated at 37 °C for 2 h, and the supernatant absorbance was measured in a microplate reader at 540 nm (OD_540_). The rate of hemolysis was calculated as shown in Eq. (2) below:

2 $$\begin{aligned} Hemolysis\;rate \left(\%\right) & = (ODexperimental groups \\&- ODnegative control group)/ \\&(ODpositive control group\\& - ODnegative control group) \times 100\%\end{aligned}$$

### Antibacterial assay against *H. pylori*

*H. pylori* were harvested in *Brucella* broth, and their density was adjusted to 10^6^ CFU/mL. The *H. pylori* suspension and 2 mL MAXPOWER Biological Antibacterial Liquid were added to a liquid culture system consisting of 5 mL *Brucella* broth, 500 µL FBS, and 25 µL mixed antibiotics (40 mg of doxycycline, 60 mg of metronidazole, 40 mg of amphotericin B, and 50 mg of vancomycin dissolved in 200 ml of sterile distilled water). The mixtures were then incubated under a 10% CO_2_ and 5% O_2_ atmosphere at 37 °C with shaking at 150 rpm for 0.5 min, 1 min, and 1.5 min. After diluting the suspension 200-fold, spread it on Columbia agar plates and then incubate for 3–4 days before counting. The absorbances of the suspensions were also measured at 600 nm (OD_600_).

### Live/dead fluorescence staining

*H. pylori* were harvested in *Brucella* broth, and their density was adjusted to 10^6^ CFU/mL. Then, 1 mL *H. pylori* was cocultured with 2 mL MAXPOWER Biological Antibacterial Liquid (a concentration of 25% of the original solution) for different lengths of time (0.5 min, 1 min, 1.5 min). Then, SYTO 9/PI Live/Dead Bacterial Double Staining Reagent was added to the suspensions, and the mixtures were incubated under low light for 15 min. Finally, the suspensions were photographed under an orthofluorescence microscope (No. MF43-N; Micro-Shot Technology (MSHOT), Guangzhou, China).

### Antibacterial mechanism study

*Helicobacter pylori* was dispersed in *Brucella* broth and incubated with PBS (control group) or MAXPOWER Biological Antibacterial Liquid (a concentration of 25% of the original solution) for 0.5 min, 1 min, and 1.5 min. Each sample was then centrifuged at 5,000 rpm for 10 min, and the supernatant was removed. ***(i) H. pylori morphological examination by scanning electron microscopy (SEM).*** The supernatant-free bacteria were fixed with 2.5% (v/v) glutaraldehyde and incubated at 4 °C overnight. The bacterial suspensions were then centrifuged at 3,000 rpm for 10 min and dehydrated in a gradient of 50% (v/v), 70% (v/v), 90% (v/v), and 100% (v/v) ethanol for 10 min/step. The samples were then freeze-dried and sputter-plated with gold. The morphology of *H. pylori* was observed under SEM, and 6 visual fields from each group were randomly selected to count the percentage of fragmentation bacteria to the total number of bacteria in that field of view. ***ii) H. pylori protein leakage***. After aspiration of the above suspension, the leaked protein concentration was measured using the BCA Kit according to the instructions provided in the manual and detected using a spectrophotometer at 562 nm. ***iii) Urease activity of H. pylori.*** The urease activity of *H. pylori* subjected to MAXPOWER Biological Antibacterial Liquid (a concentration of 25% of the original solution) for different times (0.5 min, 1 min, 1.5 min) was measured with a Urease Kit and Urease Detection Solution (0.9% (w/v) NaCl, 20 mM urea, and 14 µg/mL phenol red). The OD_550_ was measured in a spectrophotometer.

### Gut microbiota analysis and in vivo animal tissue safety assessment

Specific pathogen-free (SPF) male Kunming (KM) mice (6–7 weeks old, body weight 20–25 g) were assigned to the control and experimental groups (*n* = 6/group). The experimental group was administered MAXPOWER Biological Antibacterial Liquid (a concentration of 25% of the original solution) by gavage at a rate of 200 µL/mouse/d every morning. Gavage was administered on alternate days, and there were four gavages. The control was administered 200 µL of 0.9% saline solution. The mice had *ad libitum* food access, and their weight was recorded regularly. On the morning following the final treatment, place the mice in a clean, sterile container to collect fresh fecal samples, and the number and diversity of the bacteria in them were determined by 16 S rRNA sequencing performed at Shanghai Daixuan Biotechnology Co. (Shanghai, China). The mice were euthanized on day 28 after gavage, and their sera were collected for biochemical and routine blood tests. Hearts, livers, spleens, lungs, and kidneys were excised, dissected, and stained with hematoxylin-eosin (HE) to assess the safety of MAXPOWER Biological Antibacterial Liquid.

### Transcriptomic analysis

*Helicobacter pylori* (OD_600_ = 1) was coincubated with PBS for 2 min and MAXPOWER Biological Antibacterial Liquid (a concentration of 25% of the original solution) for 2 min, collected by centrifugation and subjected to high-throughput sequencing. The experiment utilized the TruSeq™ Stranded Total RNA Library Prep Kit (Illumina, 20,020,594) for library construction. Briefly, after removing rRNA, mRNA fragments were obtained by adding a fragmentation buffer. Subsequently, cDNA was synthesized by reverse transcription, and adaptors were ligated using End Repair Mix. Before PCR amplification, the second cDNA strand was digested using the UNG enzyme to ensure that the library only contained the first cDNA strand. Finally, sequencing was performed on the NovaSeqXPlus platform and the DNBSEQ-T7 platform. The bioinformatics data were analyzed using the Majorbio Cloud Platform (Major Biomedical Technology Co., Shanghai, China).

### Clinical study design

A prospective, randomized clinical pilot study was designed and performed per the Declaration of Helsinki, and informed consent was obtained from all patients. This study was retrospectively registered in the ClinicalTrials.gov Trial Registry on 21/09/2023 (NCT06045832). Mouthwash is a medical device and does not require ethics committee approval. The patients enrolled in the trial were oral *H. pylori*-positive and aged 18–70 y and had visited the outpatient clinic of Changhai Hospital between March 1, 2023, and August 1, 2023. The exclusion criteria were as follows: [1] Zollinger-Ellison syndrome, gastric cancer, upper gastrointestinal bleeding, or active peptic ulcer; [2] coexistence of significant concomitant illnesses including heart diseases, renal failure, hepatic diseases as well as previous abdominal surgery, lactation, or pregnancy; and [3] refusal to participate in the trial. Written informed consent was obtained from all patients before their participation.

The sample size of this study was calculated using PASS (Version11.0.7) according to Z-Test for two proportions. At least 34 patients would be required in randomized clinical pilot study based on power (1 − β), alpha (significance level), and effect size were 0.80, 0.05, and 0.45, respectively. Assuming a follow-up loss rate was 20%, the final sample size of this study was calculated as 42 patients.

At the start of the study, the demographic characteristics of all participants were recorded in detail and included sex, age, history of tobacco smoking, history of alcohol consumption, presence of halitosis, and GI symptoms. SPSS v. 25.0 (IBM Corp., Armonk, NY, USA) was used to randomize the patients to be administered either Novel MAXPOWER Biological Antibacterial Liquid (a concentration of 25% of the original solution) or water. The randomization ratio was 1:1. All patients were instructed to brush their teeth only once in the morning and before bedtime as usual for 7 days. Additionally, they were asked to rinse their mouth with Novel MAXPOWER Biological Antibacterial Liquid 3×/d for 2 min each time each time. Upon waking up on the morning of the 8th day, oral H. pylori saliva antigen (HPS) testing was immediately performed to detect oral *H. pylori* [17].

The primary outcome of the study was the oral *H. pylori* eradication rate based on intention-to-treat (ITT) and per-protocol (PP) analyses.

### Statistical analysis

All data are presented as the means ± standard deviations (SD) and were analyzed with SPSS v. 25.0 and GraphPad Prism v. 9.5 (GraphPad Software, La Jolla, CA, USA). *p* < 0.05 was considered statistically significant.

## Results

### In vitro biocompatibility and hemocompatibility

**Fig. 2:**
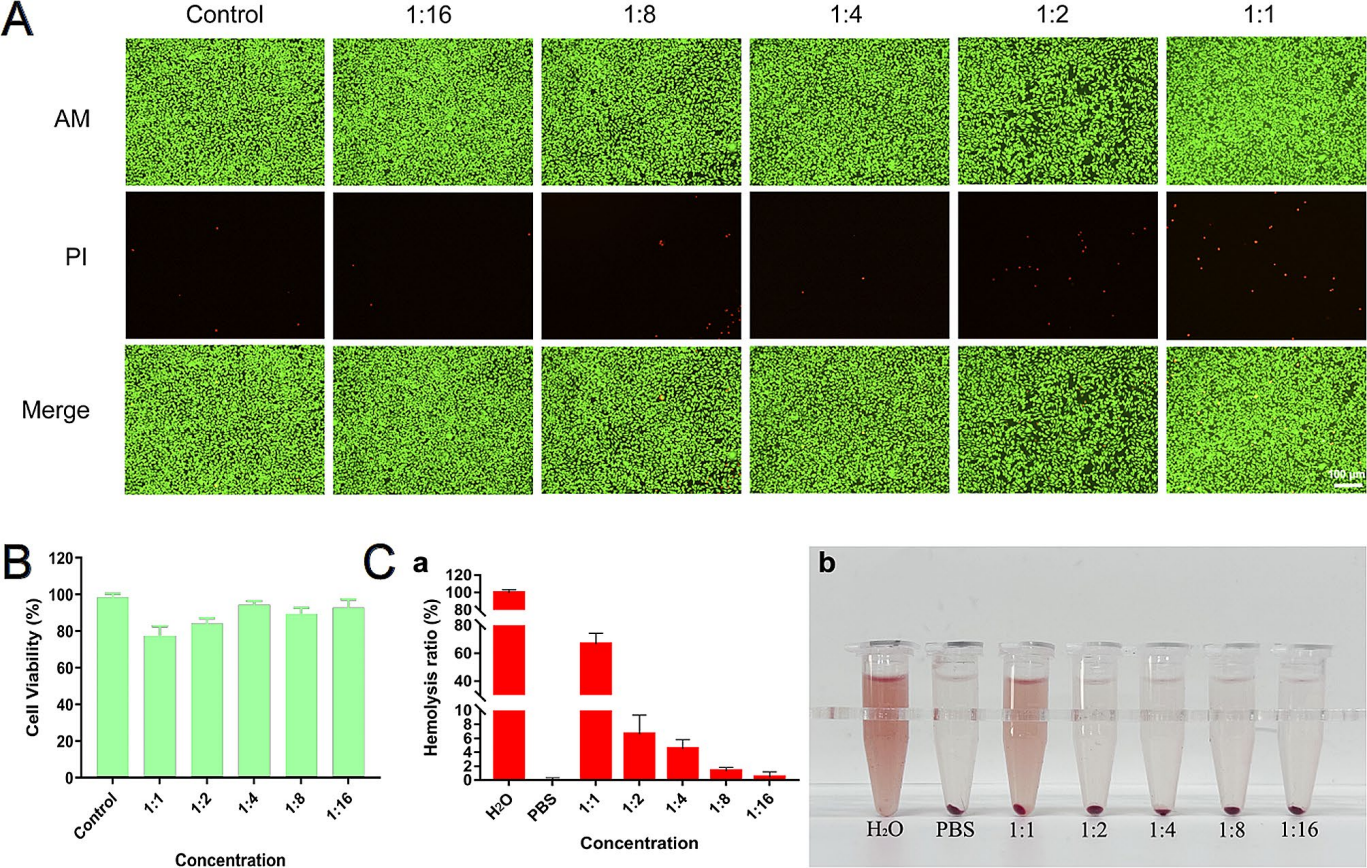
In vitro safety evaluation. (**A**) Live/dead cell staining of L929 cells after coculture with different concentrations (original solution, 1:1, 1:2, 1:4, 1:8, 1:16) of sterilized solution. (**B**) The viability of L929 cells after coculture with sterilized solution at different concentrations (original solution, 1:1, 1:2, 1:4, 1:8, 1:16). (**C**) Hemolysis rate of sterilized solution at different concentrations (original solution, 1:1, 1:2, 1:4, 1:8, 1:16) (**a**) and optical pictures (**b**). Data are presented as the mean ± SD (*n* = 3)

Biocompatibility and hemocompatibility are prerequisites for clinical product translation [18]. Here, we isocratically diluted the MAXPOWER Biological Antibacterial Liquid stock solution to screen biocompatible concentrations and used those dilutions to formulate a practical mouthwash product. L929 cells were incubated with MAXPOWER Biological Antibacterial Liquid for 24 h, and live-dead fluorescence staining assays revealed abundant dead cells (red fluorescence) at an isocratic dilution of 1:1 (c: PBS). In contrast, the L929 cells remained largely unaffected by 1:2 (MAXPOWER Biological Antibacterial Liquid: PBS) or greater, as those dilutions resulted in numerous live cells (green fluorescence) (Fig. 2A). The qualitative CCK-8 assay indicated that L929 activity levels were 84% and 94.3% at 1:2 and 1:4 (MAXPOWER Biological Antibacterial Liquid: PBS), respectively (Fig. 2B). The in vitro biocompatibility assay revealed that MAXPOWER Biological Antibacterial Liquid is biocompatible at a 1:4 dilution. The hemocompatibility assay (Fig. 2C) showed that MAXPOWER Biological Antibacterial Liquid had a hemolysis rate of 4.52% (< 5%) at a 1:4 dilution, which was close to that of PBS (0) and negligible compared to that of water (100%). Therefore, setting the concentration ratio of MAXPOWER Biological Antibacterial Liquid to 25% ensured that it would be hemocompatible. The foregoing findings indicate that MAXPOWER Biological Antibacterial Liquid is both biocompatible and hemocompatible and is safe for clinical translational use at a 1:4 dilution.

### In vitro anti-H. pylori activity

**Fig. 3:**
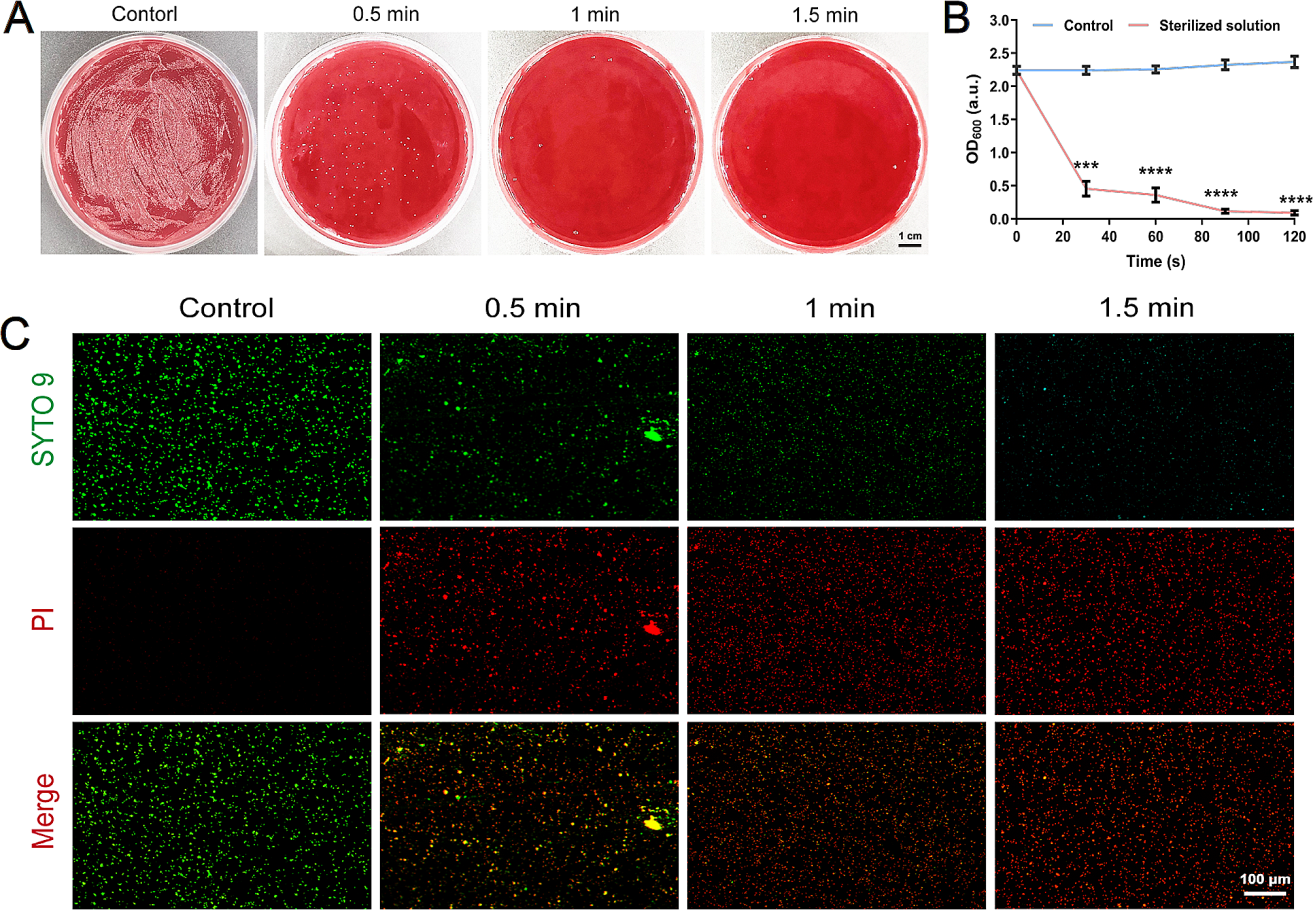
In vitro anti-H. pylori activity. Colony plate images (**A**), absorbance of bacterial liquid in OD_600_ (**B**), and live/dead bacteria staining of *H. pylori* after treatment with sterilized solution for different processing times (0.5 min, 1 min, 1.5 min). Data are presented as the mean ± SD (*n* = 3), *p < 0.05, **p < 0.01, ***p < 0.001, ****p < 0.0001

Oral *H. pylori* eradication is clinically important to improve the success rate of intragastric Helicobacter pylori eradication. The contact time between mouthwash products and *H. pylori* is short in the oral cavity. Consequently, we evaluated the in vitro efficacy with which MAXPOWER Biological Antibacterial Liquid (a concentration of 25% of the original solution) eradicates *H. pylori* within 1.5 min. Figure 3A and Supplementary Fig. 1 show that compared to the control, there was a substantial reduction in the number of *H. pylori* colonies on blood agar plates after 30 s of exposure to MAXPOWER Biological Antibacterial Liquid. After 1.5 min of contact with MAXPOWER Biological Antibacterial Liquid (a concentration of 25% of the original solution), virtually no *H. pylori* colonies remained on the blood agar plate. Hence, MAXPOWER Biological Antibacterial Liquid can rapidly kill *H. pylori*. Time-sensitive absorbance (OD_600_) measurements indicated that the *H. pylori* eradication rate varied with time after MAXPOWER Biological Antibacterial Liquid (a concentration of 25% of the original solution) addition. While the *H. pylori* eradication rate was 98.53% after 30 s of contact with MAXPOWER Biological Antibacterial Liquid, it reached 100% after 1.5 min of exposure to the product (Fig. 3B). Subsequent live/dead staining assays showed that MAXPOWER Biological Antibacterial Liquid had strong antibacterial activity. Red fluorescence was strong, and green fluorescence was weak after 1.5 min of treatment with MAXPOWER Biological Antibacterial Liquid (Fig. 3C). The preceding results suggest that MAXPOWER Biological Antibacterial Liquid effectively and rapidly eradicates *H. pylori*.

### Anti-*H. pylori* mode of action of MAXPOWER biological antibacterial liquid

**Fig. 4:**
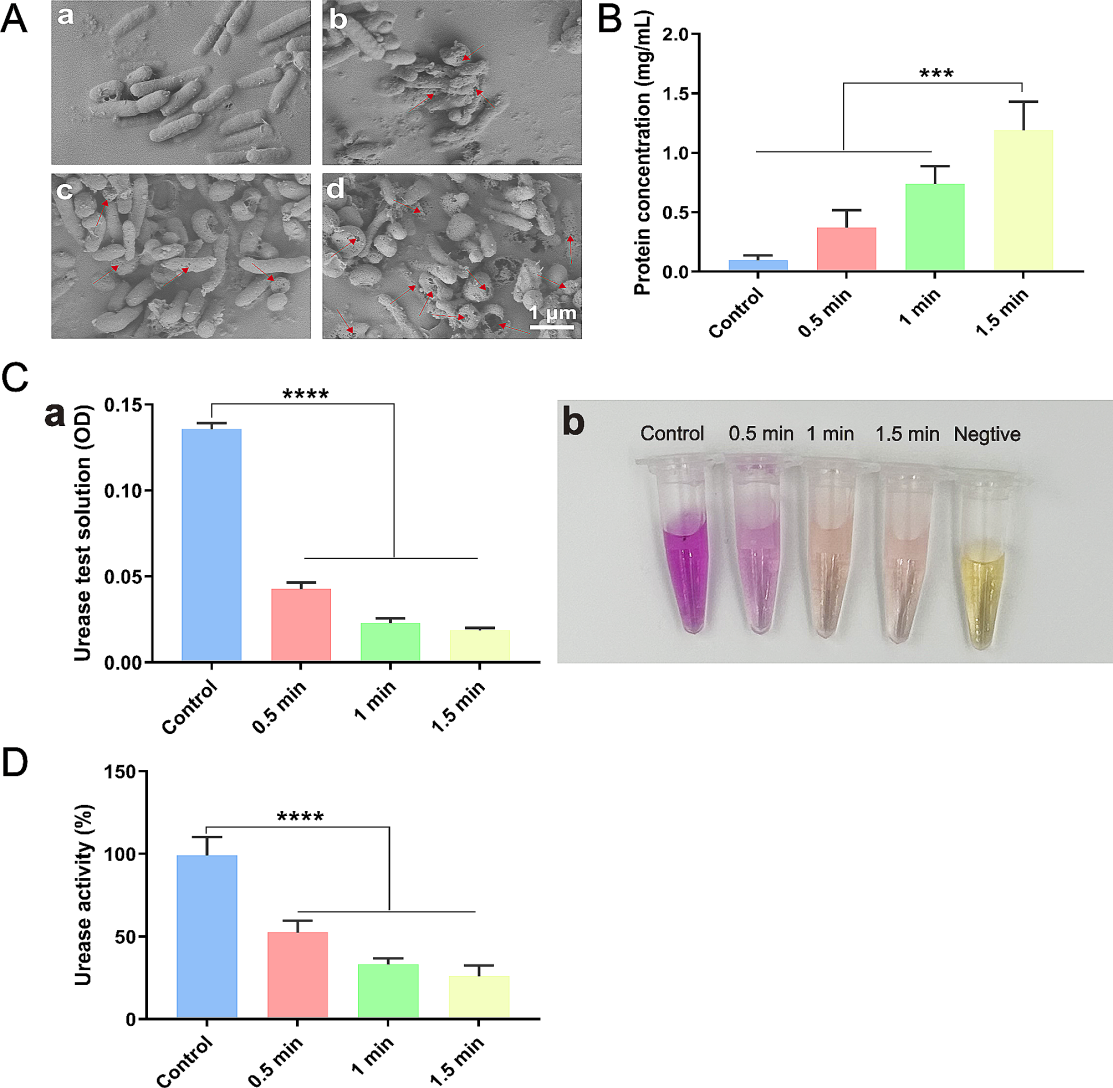
Antibacterial mechanism in vitro. (**A**) Morphological changes of *H. pylori* in SEM images after treatment with PBS (**a**) and sterilized solution for 0.5 min (**b**), 1 min (**c**), and 1.5 min (**d**). Red arrows indicate bacterial membrane rupture. (**B**) A BCA kit was used to detect the *H. pylori* overflow protein concentration after treatment with PBS and sterilized solution at different times (0.5 min, 1 min, 1.5 min). (**C**) Absorption spectra of urease detection solution (532 nm) of *H. pylori* after treatment with different samples. (**D**) The urease activity of *H. pylori* after treatment with PBS and sterilized solution at different times (0.5 min, 1 min, 1.5 min). Data are presented as the mean ± SD (*n* = 3), *p < 0.05, **p < 0.01, ***p < 0.001, ****p < 0.0001

We then investigated the mechanism by which MAXPOWER Biological Antibacterial Liquid kills *H. pylori*. Figure 4A shows the SEM structures of *H. pylori* subjected to MAXPOWER Biological Antibacterial Liquid (a concentration of 25% of the original solution) for different lengths of time (0.5 min, 1 min, 1.5 min). Compared to the control group, the surface morphology of the bacteria began to distort and deform (red arrows) after being in contact with MAXPOWER Biological Antibacterial Liquid for more than 30s. Furthermore, with an increase in contact time with MAXPOWER Biological Antibacterial Liquid, the degree of bacterial distortion became more pronounced, and the percentage of fragmented bacteria gradually increased (Supplementary Fig. 2). By that time, nearly all the *H. pylori* cells presented irregular outer membrane breakage. The results of the protein leakage assay were consistent with those of the SEM imaging (Fig. 4B). The considerable amount of protein leakage from *H. pylori* exposed to MAXPOWER Biological Antibacterial Liquid for 1.5 min confirms that the outer membranes were severely damaged by that time. MAXPOWER Biological Antibacterial Liquid also inhibited urease activity in *H. pylori*. Figure 4C shows that the color of the urease detection solution gradually decreased in intensity with increasing contact time between MAXPOWER Biological Antibacterial Liquid (a concentration of 25% of the original solution) and *H. pylori*. The red color of the urease detection solution was lightest and was nearly the same as that of the negative control after 1.5 min of exposure to the product. Hence, the *H. pylori* death rate was highest after 1.5 min of treatment (Fig. 4C). The results of the urease test corroborated those of the preceding assays (Fig. 4D). Overall, the MAXPOWER biological antibacterial liquid eradicates *H. pylori* by destroying its bacterial membrane and partially inhibits its urease activity.

### In vivo histocompatibility and intestinal flora protection

**Fig. 5:**
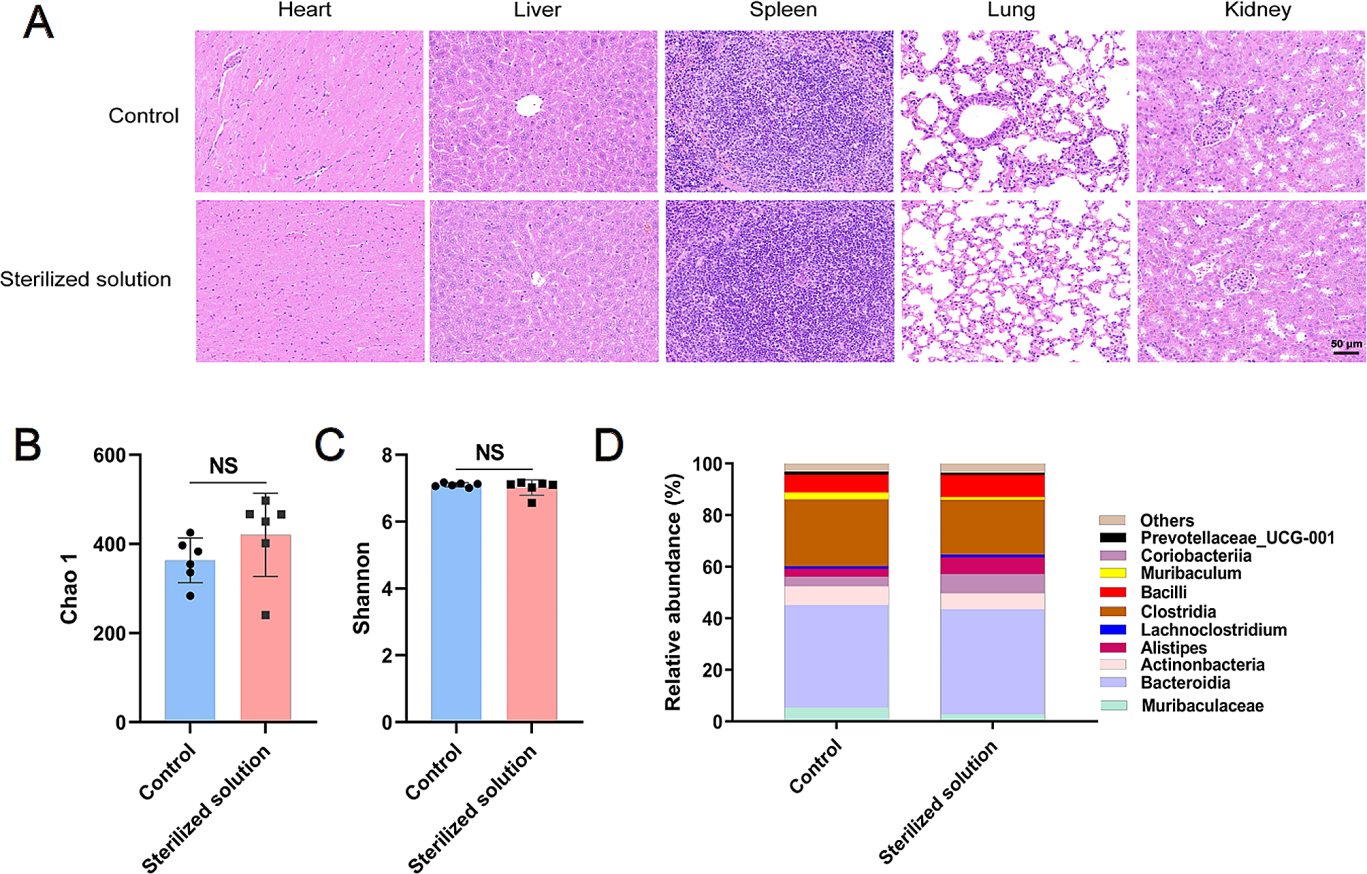
Animal safety and its impact on gut microbiota. (**A**) H&E staining results of the heart, liver, spleen, lung and kidney of mice that were treated with PBS and sterilized solution. (**B**) Chao 1 diversity and (**C**) Shannon indexes of 𝛼-diversity in the feces of mice after different treatments. (**D**) Relative abundance of colony structure in mouse feces after different treatments. Data are presented as the mean ± SD (*n* = 3), *p < 0.05, **p < 0.01, ***p < 0.001, ****p < 0.0001

Mouthwash is usually expectorated after brief contact with the oral cavity. Nevertheless, when the product is frequently used, small amounts of it may remain in the mouth and enter the stomach along with saliva. Therefore, we assessed the safety of MAXPOWER Biological Antibacterial Liquid. H&E staining of mouse liver, spleen, lung, and kidney tissues revealed that, compared with the control, the animals administered MAXPOWER Biological Antibacterial Liquid (a concentration of 25% of the original solution) by gavage for 28 d presented no obvious pathological changes (Fig. 5A). Routine tests of blood (Supplementary Fig. 3) and blood biochemistry (Supplementary Fig. 4) in mice treated with MAXPOWER Biological Antibacterial Liquid (a concentration of 25% of the original solution) were close to those of the control group. The above results suggested that MAXPOWER Biological Antibacterial Liquid has good compatibility in vivo. Conventional *H. pylori* treatments may include antibiotics that may alter the intestinal microflora and induce various diseases. We also explored any changes that MAXPOWER Biological Antibacterial Liquid (a concentration of 25% of the original solution) gavage may have caused to the mouse gut microbiota. Figure 5B and C show that there were no significant differences between the treated and control mice in terms of the α-diversity (Chao 1 and Shannon indices) of their intestinal microflora. There were also no significant differences between the treated and control mice in terms of the composition of their gut microbiota (Fig. 5D**)**. The preceding findings confirm that MAXPOWER Biological Antibacterial Liquid has good biocompatibility.

### Transcriptomic analysis of the mode of action of MAXPOWER biological antibacterial liquid against *H. pylori*

**Fig. 6:**
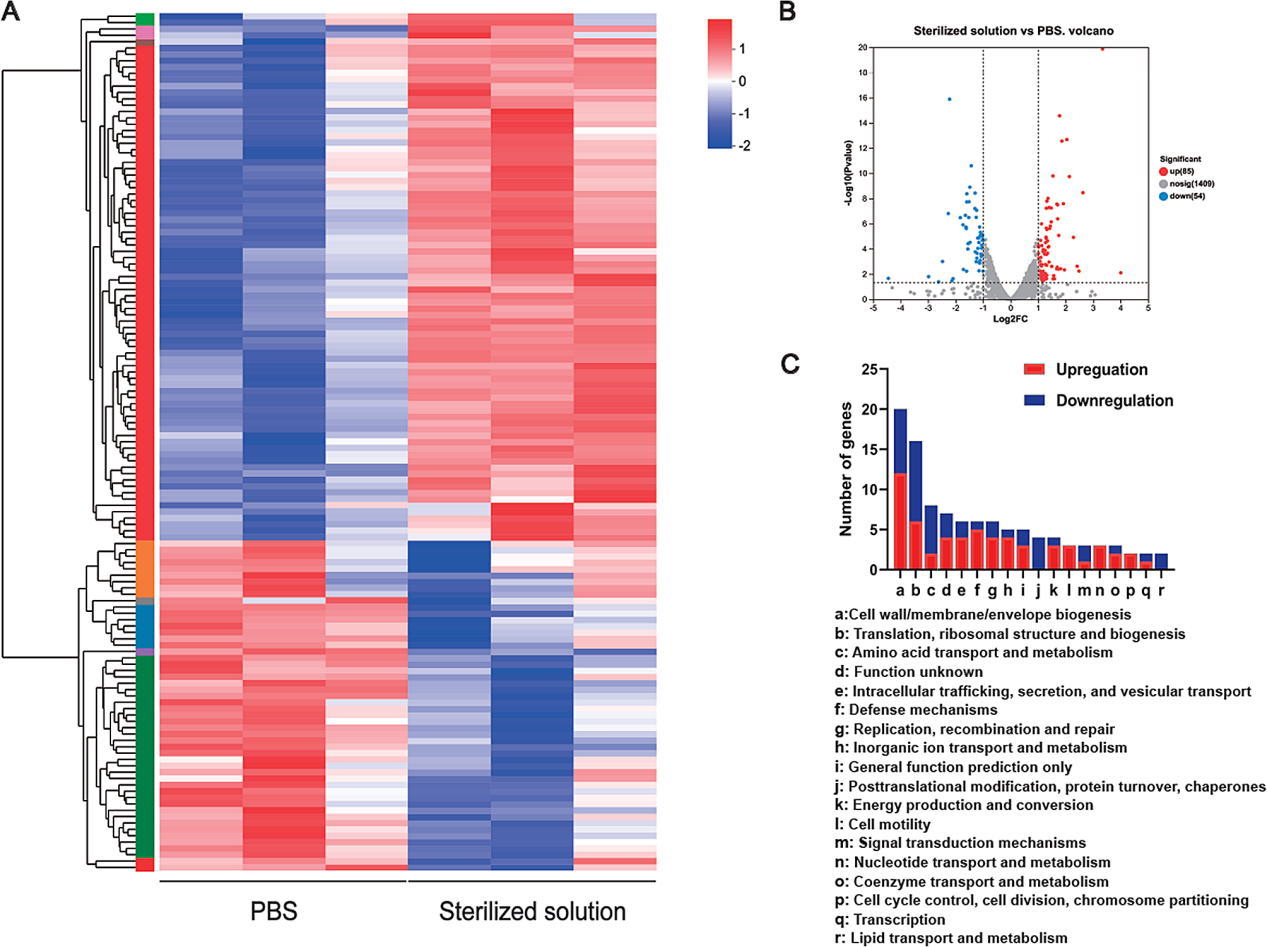
Mechanism exploration of *H. pylori* treated by MAXPOWER Biological Antibacterial Liquid. (**A**) Heatmap of the expressed genes of *H. pylori* after different treatments. (**B**) The volcano plot showing DEGs of *H. pylori* after treatment with PBS and sterilized solution. (**C**) Functional classification of the differentially expressed genes between the PBS group and sterilized solution group by COG analysis

We then performed RNA sequencing (RNA-seq) on treated *H. pylori* to determine the antibacterial mechanism of MAXPOWER Biological Antibacterial Liquid. We generated 23–32 million reads per sample and mapped them against the reference genome of the standard *H. pylori* ATCC 43,504. We identified 1,528 genes common to *H. pylori* subjected to PBS (control) and those exposed to MAXPOWER Biological Antibacterial Liquid. There were 139 differentially expressed genes (DEGs) (fold change [FC] ≥ 2 and *p* < 0.05) (Fig. 6A). Of these, 85 and 54 were significantly upregulated and downregulated, in *H. pylori* treated with sterilized solution (Fig. 6B). There were significant differences between the sterilized solution and the PBS group in terms of the genes encoding their outer membrane proteins, such as *hofD*, *hopM*, *HG583_RS03985*, and *HG583_RS02335*. Thus, brief contact with MAXPOWER Biological Antibacterial Liquid disrupted the outer membrane of *H. pylori*. The flagellum confers motility to *H. pylori* [19]. The MAXPOWER Biological Antibacterial Liquid treatment altered several genes regulating flagellar biosynthesis, including *flgE, flhF*, and *HG583_RS01290*. Therefore, MAXPOWER Biological Antibacterial Liquid modulates *H. pylori* motility by altering its flagellin biosynthesis. Moreover, the sterilized solution treatment downregulated the genes encoding translation and ribosomal structure to a greater extent than the PBS treatment. Hence, the former group had lower metabolic activity than the latter (Table S1). Figure 6C shows the functional enrichment analysis of the DEGs and indicates that the MAXPOWER Biological Antibacterial Liquid treatment attenuated bacterial membrane integrity, metabolism, defense mechanisms, and motility in *H. pylori*.

### Efficacy with which MAXPOWER biological antibacterial liquid eradicates *H. pylori* in the oral cavity

**Fig. 7:**
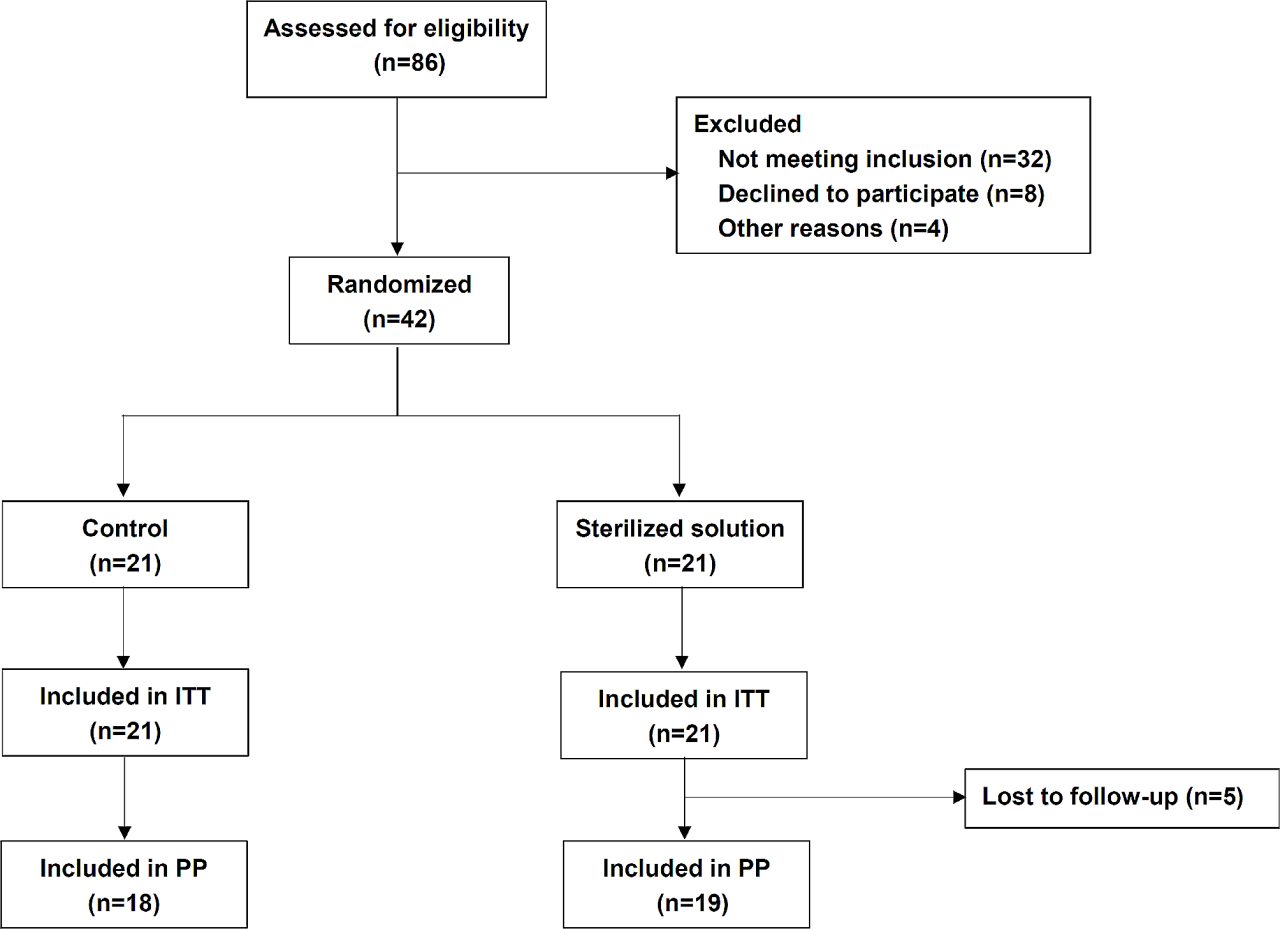
Flowchart of patients enrolled in the present study

We then designed a prospective, randomized clinical trial at Changhai Hospital to validate the efficacy with which MAXPOWER Biological Antibacterial Liquid mouthwash eradicates oral *H. pylori* in humans. Figure 7 shows the eligibility of 86 oral *H. pylori*-positive patients for the clinical trial on MAXPOWER Biological Antibacterial Liquid mouthwash. Of these, 44 were excluded because of [1] failure to meet the inclusion criteria; [2] refusal to participate; or [3] other reasons. Forty-two oral *H. pylori-*positive patients were enrolled in the clinical product efficacy trial and randomized into the MAXPOWER Biological Antibacterial Liquid gargle (sterilizing solution) and water gargle (control) groups. All patients were treated for 7 d. Three and two patients were lost from the control and sterilizing solution groups, respectively. Age, sex, tobacco and alcohol consumption, and diagnosis for each patient are listed in Table 1. There were no significant differences between groups in terms of their baseline characteristics (*p* > 0.05).

**Table 1:** Baseline of characteristics of enrolled patients in the present study

Characteristic	Control group	Sterilized solution group	*p*-value
Age (years, mean ± SD)	45.9 ± 2.4	47.8 ± 3.1	0.755
Range	25–64	29–69	
Gender (male/female)	13/8	12/9	1
Cigarette smoking	8 (38.1%)	8 (38.1%)	1
Alcohol drinking	9 (42.9%)	7 (33.3%)	0.334
Gastrointestinal symptoms	12 (57.1%)	13 (61.9%)	0.753
Abdominal distension			0.885
Abdomina pain	8 (38.1%)	9 (42.9%)	
Acid regurgitation.	5 (23.8%)	3 (14.3%)	
Paucisymptomatic	3 (14.3%)	3 (14.3)	

The relative efficacies of the oral *H. pylori* eradication treatment in the groups are shown in Table 2. The ITT analysis indicated that the oral *H. pylori* eradication rate was significantly higher in the sterilized solution group than in the control group (71.4% vs. 14.3%; *p* = 0.001). The PP analysis demonstrated that the efficacy of the sterilized solution treatment against oral *H. pylori* was significantly higher than that of the control (78.9% vs. 16.7%; *p* = 0.001). None of the patients presented any perceptible adverse reactions during treatment. The foregoing results suggest that MAXPOWER Biological Antibacterial Liquid-based mouthwash has high oral anti-*H. pylori* efficacy in humans. The main currently available methods of controlling oral *H. pylori* infection include complex periodontal cleaning and the use of antibiotic- and chlorhexidine-containing mouthwashes [20–23]. Nevertheless, the high cost of frequent periodontal scaling may hinder patient compliance, which will result in the failure to eradicate oral *H. pylori*. Furthermore, the administration of oral antibiotics can induce resistance in *H. pylori* [24]. To the best of our knowledge, the present study is one of the first to formulate and assess the efficacy of an antibiotic-free mouthwash for the eradication of oral *H. pylori*.

**Table 2:** Eradication rate of each treatment group

Analysis	Control group	Sterilized solution group	χ2	*p*-value
ITT	14.3% (3/21)	71.4% (15/21)	14	0.001
PP	16.7% (3/18)	78.9% (15/19)	14.352	0.001

## Discussion

To the best of our knowledge, the present study is one of the first to apply pure biotechnology to develop an antibiotic-free anti-*H. pylori* mouthwash and clinically assess its safety and efficacy. The results of the study indicated that the mouthwash had a favorable safety profile and effectively eradicated oral *H. pylori*.

Recently, oral *H. pylori* has attracted increasing attention, and an increasing number of studies have shown that oral *H. pylori* is homologous to gastric *H. pylori* [25–28]. Our previous national multicenter study demonstrated that the familial rate of *H. pylori* infection is high in Chinese households, as family members use the same utensils to share food and chew food before feeding children with it [15]. These results suggest that the oral cavity is an important reservoir and transmission base for *H. pylori*. Furthermore, the eradication of oral *H. pylori* may facilitate the successful eradication of gastric *H. pylori* and reduce the risk of recurring stomach infection [21, 22, 29, 30]. For example, Miyabayashi et al. performed gastric *H. pylori* eradication in oral *H. pylori-*positive and oral *H. pylori*-negative patients and found that the success rate was significantly lower in the former than in the latter (52.1% vs. 91.6%; *p* = 0028). Interestingly, after 2 years, the *H. pylori* recurrence rate was significantly higher in oral *H. pylori-*positive cases than in oral *H. pylori*-negative cases (31.5% vs. 4.2%; *p* = 0.018) [29]. Therefore, oral-gastric anti*-H. pylori* cotreatment with a mouthwash such as MAXPOWER Biological Antibacterial Liquid could effectively eradicate this infection.

MAXPOWER Biological Antibacterial Liquid contains anti-inflammatory and antibacterial substances, including caffeic acid, flavonoids, betaine, and various antibacterial peptides extracted using pure biotechnological methods. Caffeic acid is an organic acid with certain physiological activity obtained through enzymatic catalysis and microbial fermentation from natural sources. Numerous studies have confirmed the excellent antibacterial and anti-inflammatory effects of caffeic acid [31–33]. Sweet orange flavonoids, obtained through extraction techniques from green plants, are active substances with antimicrobial and antioxidant properties [34]. Flavonoids exhibit varying degrees of antibacterial activity against many microorganisms, including Gram-positive and Gram-negative bacteria, as well as several common fungi [35]. Betaine can be extracted from the roots and stems of natural plants and is used in antibacterial formulations to effectively eliminate various pathogenic bacteria [36]. These substances can be extracted from plants in nature, ensuring good biocompatibility and high antibacterial activity. Besides, as it contains no antibiotics, it is difficult for its target bacteria to develop resistance against it. Moreover, excluding or omitting antibiotics from the formulation lowers the risks of antibiotic contamination and abuse as well as the elevated healthcare costs associated with the latter [37, 38]. MAXPOWER Biological Antibacterial Liquid has hydrophilic and hydrophobic ends and an overall positive charge. We hypothesized that when the positively charged molecules in the formulation are near negatively charge*d H. pylori*, they bind the cells, penetrate their lipid membranes, increase their surface area, and destabilize their outer membranes. This mechanism occurs as the membrane of *H. pylori* lacks sufficient lipid molecules to accommodate the increase in its surface area, and the outer membrane becomes thin and porous. In this study, SEM revealed different degrees of membrane rupture in *H. pylori* exposed to MAXPOWER Biological Antibacterial Liquid. The BCA assay showed that protein leakage increased with the contact time between *H. pylori* and MAXPOWER Biological Antibacterial Liquid. Transcriptomic analysis revealed that the death of *H. pylori* subjected to MAXPOWER Biological Antibacterial Liquid was closely associated with outer membrane rupture as well as impaired metabolism, motility, and defense. This indicates that MAXPOWER Biological Antibacterial Liquid can achieve bactericidal effects by disrupting the outer membrane of *H. pylori* bacteria.

Currently, oral *H. pylori* treatment includes mouthwashes containing antibiotics, bactericidal such as chlorhexidine, complex herbal formulations, and periodontal therapy [29, 30, 39]. However, the frequent use of antibiotic-containing mouthwashes exacerbates antibiotic contamination [37]. Furthermore, mouthwashes containing chlorhexidine and other germicidal may be cytotoxic [40]. Finally, complex herbal formulations may provide only inconsistent or erratic efficacy against *H. pylori*, as they may be highly variable in quality and active ingredient content. The source materials in these herbal mixtures may widely differ depending on the collection year and harvest region. Periodontal therapy synergizes with systemic therapy in an attempt to improve the rate of gastric *H. pylori* eradication. Song et al. (2013) administered periodontal therapy twice monthly to synergize it with gastric anti-*H. pylori* therapy and showed that the former significantly reduced oral *H. pylori* infection (49.1%) [21]. Zaric et al. (2009) coadministered periodontal therapy against oral *H. pylori* and triple therapy against gastric *H. pylori*. They reported that oral *H. pylori* infection was significantly reduced (by ≤ 72.7%) after a single periodontal treatment [22]. Although periodontal therapy has proven efficacy against oral *H. pylori*, its complexity and high cost may negatively affect patient compliance and, by extension, therapeutic efficacy. Thus, the antibiotic-free mouthwash developed in the present work may effectively eradicate oral *H. pylori* and, therefore, improve the rate of successful gastric *H. pylori* eradication.

The present study had certain limitations. First, it was a single-center trial with a small sample size. Second, it did not evaluate the rate of gastric *H. pylori* eradication. Third, it did not attempt to establish whether the successful eradication of oral *H. pylori* by MAXPOWER Biological Antibacterial Liquid could also improve gastric *H. pylori* eradication.

## Conclusions

*H. pylori* has imposed a heavy socioeconomic burden on the global human population. Current research has concentrated mainly on eradicating gastric *H. pylori* infections [41]. However, traditional systemic antibiotic therapy has relatively weak efficacy against oral *H. pylori*, as it is unsuitable for the oral environment [42]. In contrast, localized oral adjuvant therapy could effectively eradicate oral *H. pylori*, improve the control of gastric *H. pylori*, and reduce the oral-oral and oral-fecal transmission of this bacterial pathogen. In the present work, we developed an antibiotic-free mouthwash consisting of simple ingredients based on MAXPOWER Biological Antibacterial Liquid formulated in China. Both in vivo and in vitro tests demonstrated the biosafety of this product and showed that it had a minimal negative impact on the intestinal microflora. In vitro tests established its antimicrobial efficacy against *H. pylori*. We used transcriptomic analysis to elucidate the mechanism of MAXPOWER Biological Antibacterial Liquid and found that oral rinsing disrupted the cell membrane integrity, metabolism, motility, and defense of *H. pylori*. We also conducted a clinical randomized controlled trial on patients who tested positive for oral *H. pylori* infection and concluded that the mouthwash was efficacious. Overall, MAXPOWER Biological Antibacterial Liquid has a good safety profile, effectively kills *H. pylori*, attenuates oral *H. pylori* colonization, and could enhance the success rate of gastric *H. pylori* eradication.

### Electronic supplementary material

Below is the link to the electronic supplementary material.

Supplementary Material 1

## Data Availability

The RNA sequences datasets generated and/or analysed during the current study are available in the Genome Sequence Archive (Genomics, Proteomics & Bioinformatics 2021) in National Genomics Data Center (Nucleic Acids Res 2022), China National Center for Bioinformation/Beijing Institute of Genomics, Chinese Academy of Sciences (GSA: CRA014535)[ https://bigd.big.ac.cn/gsa/browse/CRA014535].

## Declarations

All animal experiments were conducted per the protocols approved by the Laboratory Animal Center of Changhai Hospital of Naval Medical University (Shanghai, China) (SYXK2020-0033) and are reported in accordance with ARRIVE guidelines.

## Abbreviations

*H. pylori*: *Helicobacter pylori*
IARC: International Agency for Research on Cancer
WHO: World Health Organization
DMEM: Dulbecco’s modified Eagle’s medium
FBS: Fetal bovine serum
SPF: Specific pathogen-free
CCK-8: Cell Counting Kit-8
PI: Propidium iodide
PBS: Phosphate-buffered saline
SEM: Scanning electron microscopy
DEGs: Differentially expressed genes
