# Supplementary material for: Novel MAXPOWER biological antibacterial liquid for eradicating oral Helicobacter pylori
Source: BMC Infect Dis. 2024 May 29;24:540. doi: 10.1186/s12879-024-09424-8 (PMC11137934; doi:10.1186/s12879-024-09424-8)
Supplement: Supplementary file 1 — Supplementary Material 1 [file 12879_2024_9424_MOESM1_ESM.docx]

**Supplemental material**

**Novel MAXPOWER Biological Antibacterial Liquid for Eradicating Oral Helicobacter pylori**

Yongkang Lai^1,2^, Xiaoyang Dong^1^, Yingxiao Song^1^, Jiulong Zhao^1†^, Yiqi Du^1†^, Zhaoshen Li^1^

1 Department of Gastroenterology, Shanghai Changhai Hospital, Naval Medical University, Shanghai 200433, China

2 Department of Gastroenterology, Ganzhou People’s Hospital Affiliated to Nanchang University, Ganzhou 341000, China

* Yongkang Lai, Xiaoyang Dong and Yingxiao Song contributed equally to this paper

†Correspondence to:

Prof Yiqi Du, Department of Gastroenterology, Changhai Hospital, Naval Medical University, Shanghai 200433, China, Email: duyiqi@hotmail.com. Telephone: 021-31161353; Fax: 021-31162355.

Prof Jiulong Zhao, Department of Gastroenterology, Changhai Hospital, Naval Medical University; 168 Changhai Road, Yangpu District, Shanghai 200433, China; jlzhao9@163.com; Telephone: 021-31161354; Fax: 021-31162385;

**Supplemental figure**

FigS. 1 Colony count plots of *H. pylori* after treatment with sterilized solution.


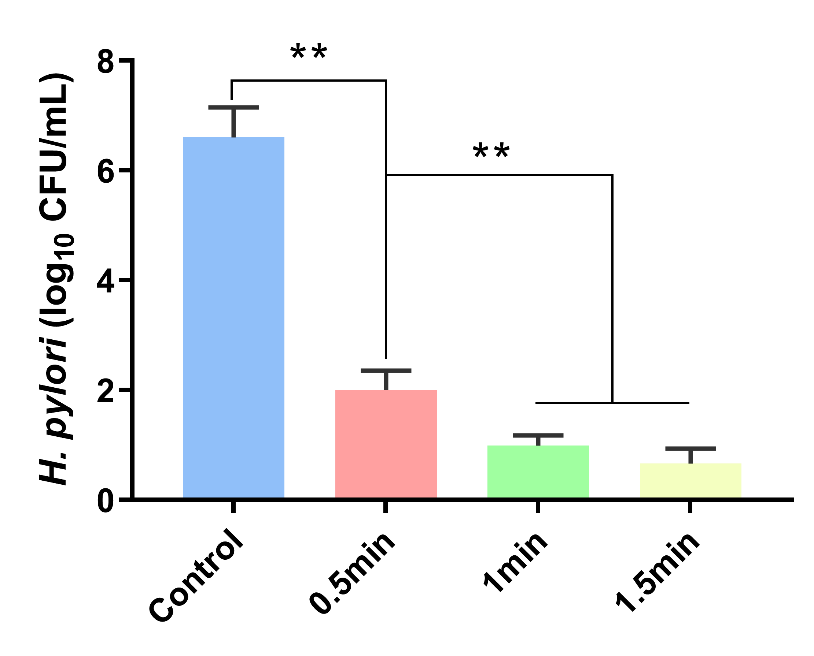


FigS. 2 Percentage of fragmented bacteria in the field of view of SEM. 6 visual fields from each group were randomly selected to count percentage of fragmentation bacteria to the total number of bacteria in that field of view. Data are presented as the mean ± SD (n = 10), **p <*0.05, ***p <*0.01, ****p <*0.001, *****p <*0.0001.


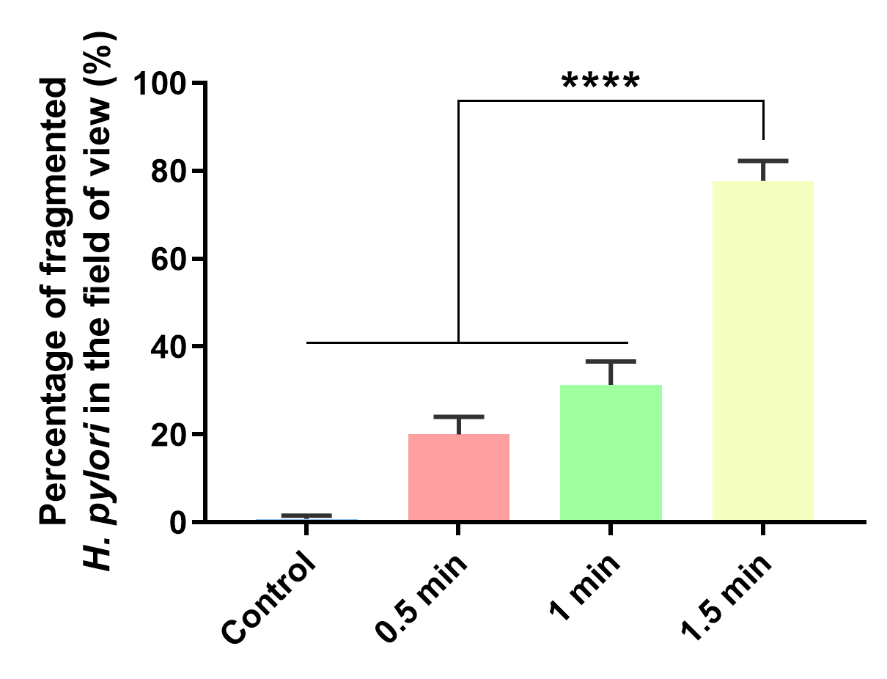


FigS. 3 Routine blood tests in mice after sterilized solution treatment.


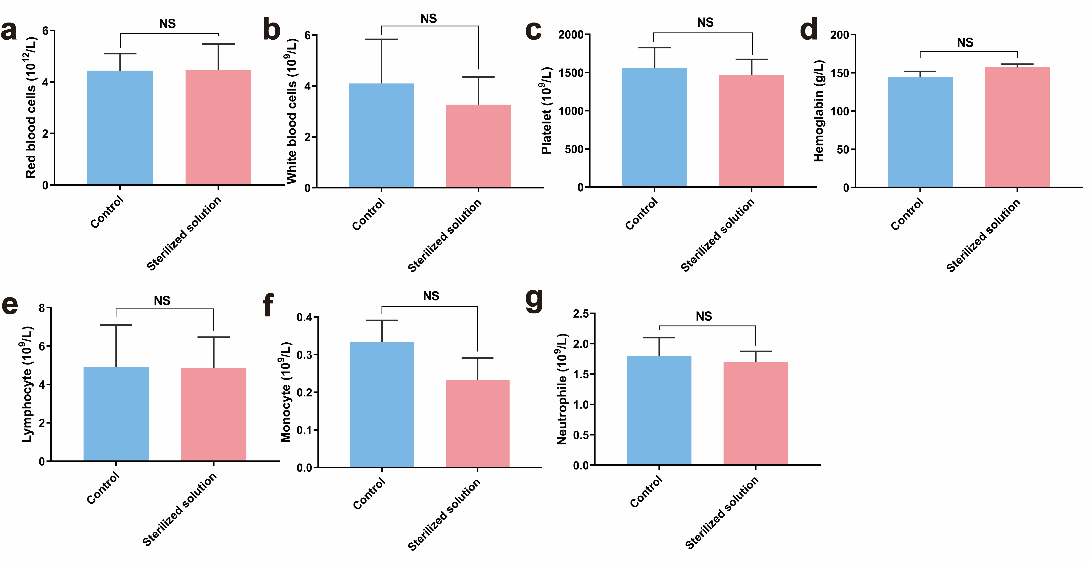


FigS. 4 Functional assay of liver and kidney function in mice after sterilized solution treatment.
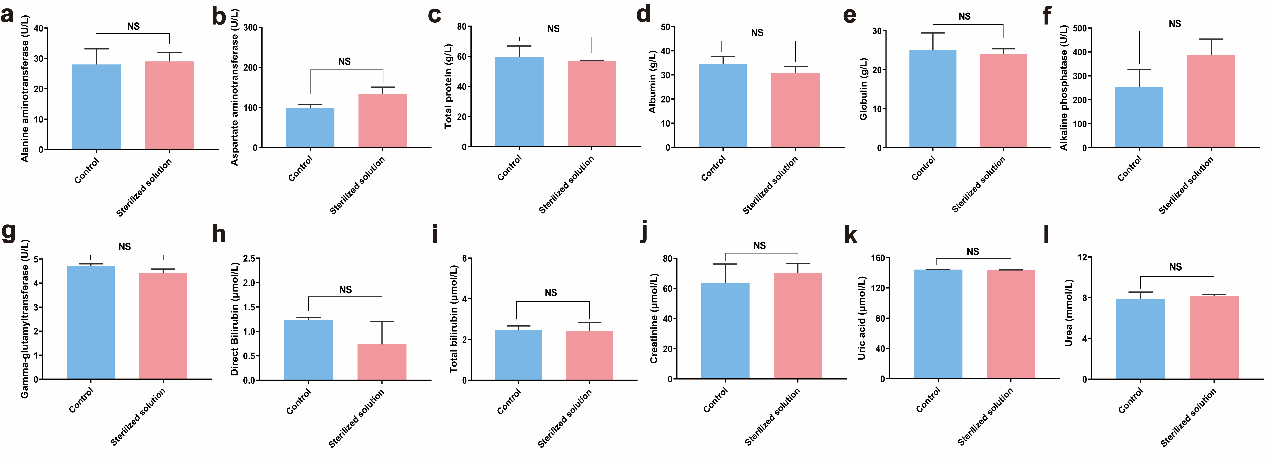


**Supplemental table**

| **Table S1 Differentially expressed genes in sterilized solution group using RNA-seq analysis** | | | |
| --- | --- | --- | --- |
| **Regulation** | **Gene name** | **Gene description** | **Log2 (Fold change)** |
| Up | ybeY | rRNA maturation RNase YbeY | 3.342463974 |
|  | rho | transcription termination factor Rho | 1.77712576 |
|  | flgE | flagellar hook protein FlgE | 2.043087183 |
|  | HG583_RS03985 | outer membrane protein | 1.859946944 |
|  | hofD | outer membrane beta-barrel protein HofD | 1.536334507 |
|  | HG583_RS07875 | exodeoxyribonuclease III | 2.132321235 |
|  | murI | glutamate racemase | 2.627233153 |
|  | HG583_RS01245 | DEAD/DEAH box helicase | 1.355602326 |
|  | HG583_RS00540 | O-acetylserine-dependent cystathionine beta-synthase | 1.315168423 |
|  | prfA | peptide chain release factor 1 | 1.915709996 |
|  | yidC | membrane protein insertase YidC | 1.680392977 |
|  | HG583_RS00100 | VirB4 family type IV secretion/conjugal transfer ATPase | 1.704617819 |
|  | hopQ | Hop family adhesin HopQ | 1.394870138 |
|  | HG583_RS00205 | virB8 family protein | 1.470593092 |
|  | atpG | ATP synthase F1 subunit gamma | 1.298928525 |
|  | waaA | lipid IV(A) 3-deoxy-D-manno-octulosonic acid transferase | 1.707700837 |
|  | HG583_RS01915 | ABC transporter ATP-binding protein | 1.473394731 |
|  | HG583_RS05425 | 30S ribosomal protein S1 | 1.117428465 |
|  | dprA | DNA-processing protein DprA | 1.43767232 |
|  | HG583_RS07355 | ribonuclease J | 1.298621273 |
|  | rsmH | 16S rRNA (cytosine(1402)-N(4))-methyltransferase RsmH | 1.422219035 |
|  | HG583_RS04250 | RelA/SpoT family protein | 1.318410713 |
|  | HG583_RS05175 | dynamin-like GTPase family protein | 1.746903866 |
|  | HG583_RS07110 | prephenate dehydrogenase | 1.28354044 |
|  | yidD | membrane protein insertion efficiency factor YidD | 2.281399897 |
|  | HG583_RS07350 | KpsF/GutQ family sugar-phosphate isomerase | 1.290981546 |
|  | HG583_RS01250 | prohibitin family protein | 1.084075621 |
|  | HG583_RS00090 | TrbC/VirB2 family protein | 1.338120918 |
|  | HG583_RS06625 | glycosyltransferase family 9 protein | 1.268953318 |
|  | flhF | flagellar biosynthesis protein FlhF | 1.002502938 |
|  | purA | adenylosuccinate synthase | 1.13031299 |
|  | cag4 | VirB1 family T4SS lytic transglycosylase Cag4 | 1.384022463 |
|  | HG583_RS05855 | FoF1 ATP synthase subunit B' | 1.012637039 |
|  | HG583_RS05795 | hypothetical protein | 1.016344981 |
|  | HG583_RS05385 | molybdopterin guanine dinucleotide-containing S/N-oxide reductase | 1.366298791 |
|  | fmt | methionyl-tRNA formyltransferase | 1.182762262 |
|  | HG583_RS02305 | TerB family tellurite resistance protein | 1.254797656 |
|  | HG583_RS00215 | DNA type IV secretion system protein ComB10 | 1.175975613 |
|  | HG583_RS00210 | TrbG/VirB9 family P-type conjugative transfer protein | 1.339785415 |
|  | HG583_RS06350 | aspartate kinase | 1.007067045 |
|  | HG583_RS07825 | aminotransferase class V-fold PLP-dependent enzyme | 1.230783122 |
|  | HG583_RS05125 | RluA family pseudouridine synthase | 1.236965098 |
|  | pseC | UDP-4-amino-4%2C6-dideoxy-N-acetyl-beta-L-altrosamine transaminase | 1.300209333 |
|  | HG583_RS04170 | Na+/H+ antiporter family protein | 1.095846757 |
|  | HG583_RS06345 | RNA pyrophosphohydrolase | 1.023040822 |
|  | HG583_RS02135 | O-antigen ligase | 1.002195799 |
|  | HG583_RS08245 | glycosyltransferase family 4 protein | 1.065134218 |
|  | HG583_RS06355 | HobA family DNA replication regulator | 1.392696191 |
|  | HG583_RS03235 | radical SAM/SPASM domain-containing protein | 1.265334907 |
|  | clsC | cardiolipin synthase ClsC | 1.133812693 |
|  | rplT | 50S ribosomal protein L20 | 1.086323272 |
|  | HG583_RS04385 | DUF3943 domain-containing protein | 1.304905436 |
|  | HG583_RS05195 | TolC family protein | 1.114397939 |
|  | HG583_RS01805 | RluA family pseudouridine synthase | 2.413658405 |
|  | HG583_RS01295 | MotE family protein | 1.493630135 |
|  | HG583_RS06295 | RDD family protein | 1.691504541 |
|  | HG583_RS04230 | hypothetical protein | 1.815346812 |
|  | HG583_RS06360 | DNA polymerase III subunit delta' | 1.673570604 |
|  | HG583_RS01290 | flagellar export protein FliJ | 1.731746417 |
|  | trmD | tRNA (guanosine(37)-N1)-methyltransferase TrmD | 1.954197318 |
|  | HG583_RS05330 | ferrochelatase | 1.004367976 |
|  | HG583_RS03310 | hypothetical protein | 1.055438288 |
|  | sRNA0073 |  | 2.480940169 |
|  | sRNA0055 |  | 2.480940169 |
|  | HG583_RS01455 | hypothetical protein | 1.176535827 |
|  | rimM | ribosome maturation factor RimM | 1.10288697 |
|  | HG583_RS03410 | 3-methyladenine DNA glycosylase | 1.143177104 |
|  | HG583_RS03925 | hypothetical protein | 4.001468522 |
|  | eptA | phosphoethanolamine--lipid A transferase EptA | 1.265736214 |
|  | bioD | dethiobiotin synthase | 1.180056792 |
|  | HG583_RS01920 | neuraminyllactose-binding hemagglutinin family protein | 1.308451651 |
|  | HG583_RS08475 | CMP-N-acetylneuraminic acid synthetase | 1.063116034 |
|  | HG583_RS05810 | energy transducer TonB | 1.565683037 |
|  | HG583_RS06950 | SH3 domain-containing protein | 1.188147095 |
|  | lpxB | lipid-A-disaccharide synthase | 1.12590067 |
|  | HG583_RS02390 | DNA cytosine methyltransferase | 1.113006449 |
|  | HG583_RS03285 | LTA synthase family protein | 1.27856027 |
|  | murA | UDP-N-acetylglucosamine 1-carboxyvinyltransferase | 1.034759606 |
|  | HG583_RS05575 | restriction endonuclease | 1.300141947 |
|  | HG583_RS05815 | ExbD/TolR family protein | 1.589927468 |
|  | HG583_RS07245 | Jag N-terminal domain-containing protein | 1.541927718 |
|  | HG583_RS02300 | motility associated factor glycosyltransferase family protein | 1.205314374 |
|  | HG583_RS03400 | ABC transporter ATP-binding protein | 1.125537771 |
|  | infA | translation initiation factor IF-1 | 1.222489827 |
|  | HG583_RS07410 | UDP-N-acetylmuramate dehydrogenase | 1.158664329 |
| Down | HG583_RS05780 | flagellar biosynthesis anti-sigma factor FlgM | -2.220191196 |
|  | lpxC | UDP-3-O-acyl-N-acetylglucosamine deacetylase | -1.435285434 |
|  | sodB | superoxide dismutase [Fe] | -1.485612494 |
|  | HG583_RS02335 | outer membrane beta-barrel protein | -1.293583057 |
|  | exbB | TonB-system energizer ExbB | -1.594447675 |
|  | HG583_RS05600 | ribonucleotide-diphosphate reductase subunit beta | -1.516929471 |
|  | HG583_RS06385 | DUF507 family protein | -1.597107151 |
|  | cagG | cag pathogenicity island type IV secretion system translocation protein CagG | -1.299379543 |
|  | HG583_RS05510 | M48 family metallopeptidase | -1.241197453 |
|  | HG583_RS07900 | DUF2443 domain-containing protein | -2.268245502 |
|  | HG583_RS05050 | RidA family protein | -1.644871245 |
|  | HG583_RS01695 | HugZ family heme oxygenase | -1.255475689 |
|  | HG583_RS07985 | hypothetical protein | -1.525527479 |
|  | HG583_RS03460 | hypothetical protein | -1.832682761 |
|  | ccoS | cbb3-type cytochrome oxidase assembly protein CcoS | -1.732019012 |
|  | HG583_RS03000 | hypothetical protein | -1.130178285 |
|  | HG583_RS02950 | hypothetical protein | -1.623478413 |
|  | HG583_RS06320 | rhodanese-like domain-containing protein | -1.612691254 |
|  | HG583_RS00660 | 3-deoxy-7-phosphoheptulonate synthase class II | -1.096676282 |
|  | HG583_RS05030 | amino acid ABC transporter substrate-binding protein | -1.023300229 |
|  | HG583_RS05755 | HP1117 family Sel1-like repeat protein | -1.098313788 |
|  | HG583_RS06850 | efflux RND transporter permease subunit | -1.029883645 |
|  | HG583_RS07925 | type I restriction endonuclease subunit R | -1.16168364 |
|  | trpS | tryptophan--tRNA ligase | -1.198416376 |
|  | hopM | Hop family outer membrane protein HopM/HopN | -1.160252235 |
|  | HG583_RS06140 | hypothetical protein | -1.001066687 |
|  | panB | 3-methyl-2-oxobutanoate hydroxymethyltransferase | -1.032091592 |
|  | HG583_RS04135 | FlaG family protein | -1.478522405 |
|  | HG583_RS00655 | serine/threonine transporter | -1.157534353 |
|  | modA | molybdate ABC transporter substrate-binding protein | -1.546990637 |
|  | HG583_RS06915 | outer membrane beta-barrel protein | -1.054030597 |
|  | HG583_RS04290 | hypothetical protein | -1.060490693 |
|  | HG583_RS05500 | SPOR domain-containing protein | -1.195686344 |
|  | HG583_RS01685 | type II toxin-antitoxin system antitoxin | -1.557607126 |
|  | rpsU | 30S ribosomal protein S21 | -1.286129212 |
|  | HG583_RS00725 | DUF4006 family protein | -1.217150424 |
|  | csd3 | peptidoglycan DD-metalloendopeptidase Csd3 | -1.074315592 |
|  | HG583_RS03260 | hypothetical protein | -1.066819146 |
|  | HG583_RS06030 | amino acid ABC transporter ATP-binding protein | -1.226584852 |
|  | HG583_RS01480 | YfhL family 4Fe-4S dicluster ferredoxin | -1.101174444 |
|  | sRNA0025 |  | -2.476816891 |
|  | pyrF | orotidine-5'-phosphate decarboxylase | -1.255550241 |
|  | HG583_RS08365 | glycosyltransferase family 25 protein | -1.158901809 |
|  | HG583_RS01220 | DUF2018 family protein | -1.147005486 |
|  | HG583_RS06040 | hypothetical protein | -1.127436894 |
|  | HG583_RS06815 | hypothetical protein | -1.71958008 |
|  | HG583_RS01820 | PAP2 family protein | -1.149004703 |
|  | HG583_RS07845 | class I SAM-dependent DNA methyltransferase | -1.005411608 |
|  | HG583_RS04430 | class I SAM-dependent methyltransferase | -1.604260931 |
|  | sRNA0030 |  | -2.983843832 |
|  | HG583_RS08335 | hypothetical protein | -4.445123028 |
|  | HG583_RS03005 | DNA topoisomerase | -2.094437259 |
|  | HG583_RS01090 | beta-1%2C4-N-acetylgalactosaminyltransferase | -2.136005121 |
|  | HG583_RS04160 | hypothetical protein | -2.621191912 |
